# Genome-wide transcriptome analysis of Chinese pollination-constant nonastringent persimmon fruit treated with ethanol

**DOI:** 10.1186/1471-2164-15-112

**Published:** 2014-02-08

**Authors:** Chun Luo, Qinglin Zhang, Zhengrong Luo

**Affiliations:** 1 Key Laboratory of Horticultural Plant Biology (MOE), Huazhong Agricultural University, 430070 Wuhan, China; 2 Key Laboratory of Tropical Fruit Biology, Ministry of Agriculture, South Subtropical Crops Research Institute, Chinese Academy of Tropical Agricultural Sciences, Zhanjiang 524091, China

**Keywords:** Persimmon, Transcriptome analysis, 454 sequencing

## Abstract

**Background:**

The persimmon *Diospyros kaki* Thunb. is an important commercial and deciduous fruit tree. The fruits have proanthocyanidin (PA) content of >25% of the dry weight and are astringent. PAs cause astringency that is often undesirable for human consumption; thus, the removal of astringency is an important practice in the persimmon industry. Soluble PAs can be converted to insoluble PAs by enclosing the fruit in a polyethylene bag containing diluted ethanol. The genomic resource development of the persimmon is delayed because of its large and complex genome. Second-generation sequencing is an efficient technique for generating huge sequences that can represent a large number of genes and their expression levels.

**Results:**

We used 454 sequencing for the *de novo* transcriptome assembly of persimmon fruit treated with 5% ethanol (Tr library) and without treatment as the control (Co library) to investigate the genes and pathways that control PA biosynthesis and other secondary metabolites. We obtained 374.6 Mb in clean nucleotides comprising 624,690 and 626,203 clean sequencing reads from the Tr and Co libraries, respectively. We also identified 83,898 unigenes; 54,719 (~65.2%) unigenes were annotated based on similarity searches with known proteins. Up to 14,954 of the unigenes were assigned to the protein database Clusters of Orthologous Groups (COG), 24,337 were assigned to the term annotation database of Gene Ontology (GO), and 45,506 were assigned to 200 pathways in the database of Kyoto Encyclopedia of Genes and Genomes (KEGG). The two libraries were compared to identify the differentially expressed unigenes. The expression levels of genes involved in PA biosynthesis and tannin coagulation were analysed, and some of them were verified using quantitative real time PCR (qRT-PCR).

**Conclusions:**

This study provides abundant genomic data for persimmon and offers comprehensive sequence resources for persimmon research. The transcriptome dataset will improve our understanding of the molecular mechanisms of tannin coagulation and other biochemical processes in persimmons.

## Background

The persimmon *Diospyros kaki* Thunb. (2n = 6X = 90) originated in China and was principally cultivated in China, Korea and Japan [1]. Persimmon cultivars are classified into four types, including pollination-constant nonastringent (PCNA), pollination-constant astringent (PCA), pollination-variant nonastringent (PVNA), and pollination-variant astringent (PVA); this classification is based on the effect of pollination on flesh colour and the natural loss of astringency at the harvest time on the tree [1]. The PCNA type includes Japanese PCNA (JPCNA) and Chinese PCNA (CPCNA), which differ in their genetic characteristic of PCNA trait [2]. The natural loss of astringency is a trait that is qualitatively inherited and recessive in JPCNA cultivars [3,4] but dominant in CPCNA cultivars. When the CPCNA cultivar ‘Luotian-tianshi’ is crossed as the maternal parent to a JPCNA or non-PCNA type, the F_1_ offspring are segregated into a 1:1 ratio for PCNA:non-PCNA types [5,6]. CPCNA cultivars have attracted attention in the breeding industry because of their natural ability to lose astringency, which is a dominant trait. In addition, CPCNA has the potential to be an important parent in PCNA persimmon breeding in the future.

Persimmon resources are widely distributed in China. However, almost all traditional cultivars native to China are of the PCA type; some of these cultivars include ‘Mopanshi’, ‘Fuping-jianshi’, and ‘Gongcheng-shuishi’ [7]. ‘Luotian-tianshi’ (*D. kaki* Thunb.; 2n = 6X = 90) is the first PCNA persimmon native to China, and it is only distributed in Dabieshan Mountain around the junction of Hubei, Henan, and Anhui provinces in central China [4,8].

Most persimmon fruits accumulate proanthocyanidins (PAs) in their flesh during development, causing the sensation of astringency due to the coagulation of oral proteins [9]. PAs or condensed tannins are synthesised *via* the shikimate and flavonoid biosynthetic pathways [10-12]. To date, many genes encoding the structural proteins and transcription factors involved in PA biosynthesis, transportation, and polymerisation have been isolated by homology-based cloning [13-23]. However, the primary genes involved in PA biosynthesis have not yet been determined.

High-throughput sequencing technologies developed in recent years provide a convenient way of establishing a rapid and efficient molecular research platform. Next-generation sequencing (NGS) is related to the Sanger sequencing method, which is represented by first-generation sequencing technologies. Currently, the three mainstream NGS technologies are Roche/454 pyrosequencing (developed in 2005, http://www.454.com), Illumina/Solexa sequencing (developed in 2006, http://www.illumina.com), and ABI/SOLiD sequencing (developed in 2007, http://www.appliedbiosystems.com). These NGS technologies vary in their input requirements and sequence output with regard to the total bases sequenced, length of each sequence read, and price per megabase of sequence information [24]. Among these technologies, 454 sequencing, which generates a minimum number of sequence reads, produces the longest reads (i.e. from 100 bp to ~800–1000 bp). Long reads are optimal for initial genome and transcriptome characterisation because longer pieces are assembled more efficiently than shorter pieces [25]. Given their rapid processing, high throughput, and cost effectiveness, NGS technologies have been successfully used to study genomes and transcriptomes of species with and without sequenced genomes. Many novel and functional genes can be obtained from massive amounts of data.

Abundant genetic resources for persimmons are currently available. However, genomic information and EST sequences for this fruit tree are lacking. In addition, molecular data on persimmons are insufficient when compared to those of other fruit trees, such as apple, pear, peach, citrus, and grape. Accordingly, we performed large-scale transcriptome sequencing of CPCNA persimmon fruit using Roche/454 technology to create a transcript sequence database of the persimmon and identify candidate genes involved in PA biosynthesis and tannin coagulation. We used IDEG6 to filter the differentially expressed genes in the treatment (Tr) and control (Co) libraries. We also verified the differentially expressed unigenes by quantitative real time PCR (qRT-PCR). The present study provides a platform for studying the genes involved in persimmon tannin coagulation and tannin biosynthesis to analyse the relationship between differentially expressed genes and persimmon fruit deastringency and clarify the mechanism of astringency loss for CPCNA.

## Results

### Sequencing and assembly

**Figure 1:**
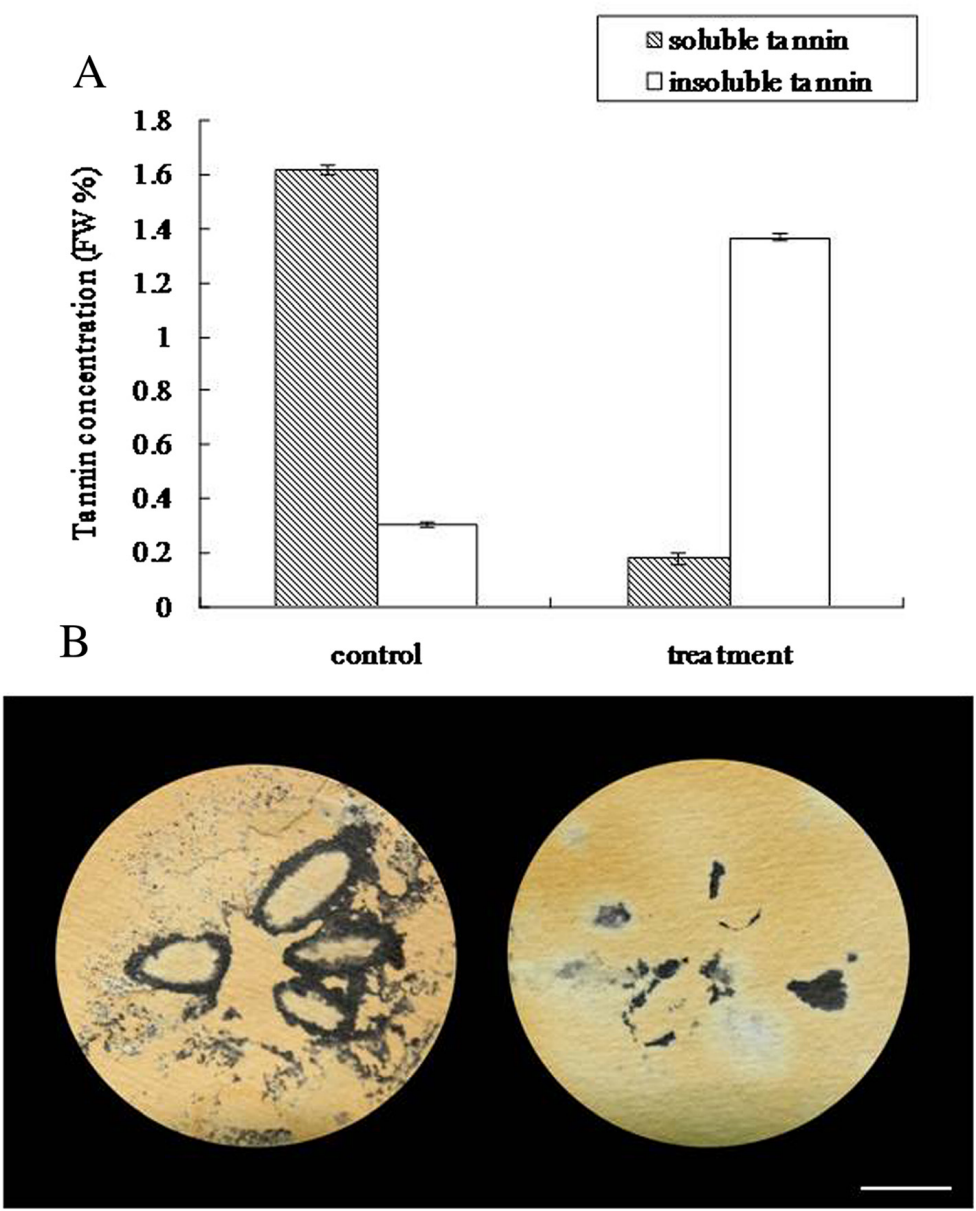
**Effect of ethanol treatment on deastringency. A**: Effect of ethanol treatment on soluble tannin content and insoluble tannin content of ‘Luotian-tianshi’. FW% means the tannin concentration per fresh weight. When it is lower than 0.2%, the persimmon fruit is not astringent. **B**: Analysis of soluble tannin content by FeCl_2_ blot. FeCl_2_ reacts with the soluble PAs, and the darker the resulting product is, the more astringent the fruit are. Bar = 1 cm.

**Figure 2:**
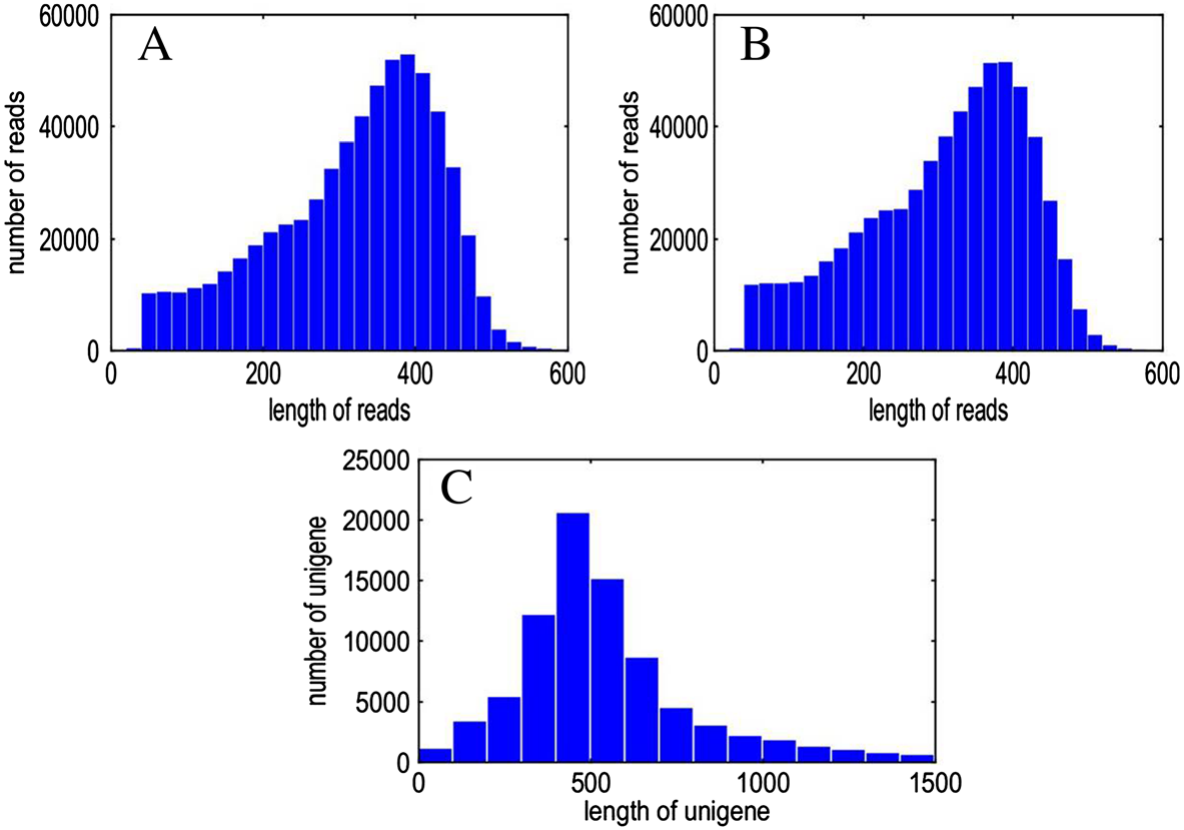
**Frequency distribution of 454 sequencing read lengths for treatment (Tr) library (A), control (Co) library (B) and assembled unigenes (C).** Tr library, Co library, and assembled unigenes with an average sequence length of 319, 309 and 579 base pairs, respectively.

The soluble PA concentration of the CPCNA persimmon fruit was <0.2% in the group treated with 5% ethanol after 3 d but was still very high (1.4%) in the control group (Figure 1A), suggesting that astringency was successfully removed in the treated fruit. In the printing test, the colour change observed after the reaction between FeCl2 and soluble tannin was dark in the control group but light in the treatment group, suggesting that the amount of soluble tannin decreased in the treated fruit (Figure 1B). A half-plate run using the 454 GS FLX Titanium platform was carried out on the cDNA that was generated by SMART technology with the total RNA from the Tr (5% ethanol-treated) and Co libraries. A total of 624,690 and 626,203 high-quality reads were generated from the Tr and Co libraries, with average sequence lengths of 319 and 309 bp, respectively [National Center for Biotechnology Information (NCBI) Short Read Archive, accession SRA091427]. After trimming the adaptor sequences and removing those shorter than 100 bp, the clean reads of the two libraries were assembled into 83,898 unique sequences using Mimicking Intelligent Read Assembly (MIRA), with an average size of 579 bp. A summary of the 454 sequencing and assembly is presented in Table 1, and the size distributions for these reads and unigenes are presented in Figure 2.

**Table 1:** Summary of the sequencing and assembly

	**Treatment library**	**Control library**
No. of HQ reads	624,690	626,203
Total nucleotides (nt)	199,375,526	193,382,890
Mean length of read (bp)	319	309
No. of unigenes of combined data	83,898	
Total nucleotides (nt) of unigenes	48,556,834	
Mean length of unigenes (bp)	579	
No. of contigs above 500 bp	41,451	

### Sequence annotation

Estimating the number of genes and the level of transcript coverage is difficult because of the lack of genetic or genomic information on persimmons. We performed BLASTX alignments (E-value < 10^−5^ or 10^−10^) against the databases of NCBI-nonredundant (Nr), SwissProt, Gene Ontology (GO), and Kyoto Encyclopedia of Genes and Genomes (KEGG) to identify the putative functions of the unigene sequences. A total of 54,719 unigenes, accounting for 65.2% of the total unigenes, were annotated to Nr database (Table 2).

**Table 2:** Annotation of non-redundant unigenes

**Database**	**Number of annotated unigenes**	**Percentage of annotated unigenes (%)**
Nr	54,719	65.2
Swissprot	31,390	37.4
KEGG	31,211	37.2
COG	14,954	17.8
Interpro	33,287	39.7
GO	24,337	29.0

**Figure 3:**
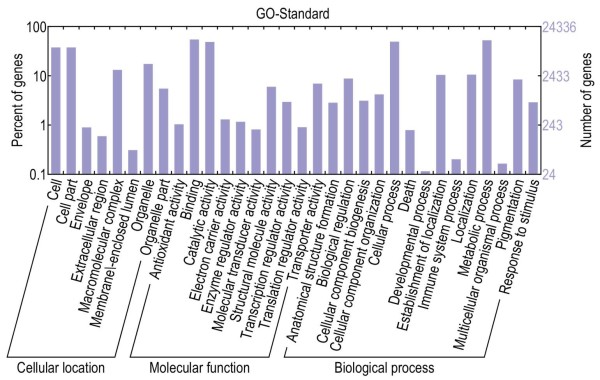
Gene Ontology classifications of assembled non-redundant unigenes.

We used GO to obtain a functional annotation of the persimmon unigenes [26]. A total of 24,337 unigenes (29.0%) were assigned to at least one GO term (Figure 3). Of the unigenes assigned to GO terms, 27,406 were assigned to cellular location, 30,629 to molecular function, and 39,037 to biological processes (Additional file 1). These unigenes were further classified into 47 functional subcategories. Within each of the three main categories of the GO classification scheme (i.e. cellular location, molecular function, and biological process), the dominant subcategories were ‘cell’ (9106), ‘cell part’ (9106), ‘binding’ (13,212), ‘catalytic activity’ (11,774), ‘cellular process’ (11,929), and ‘metabolic process’ (12,712).

**Figure 4:**
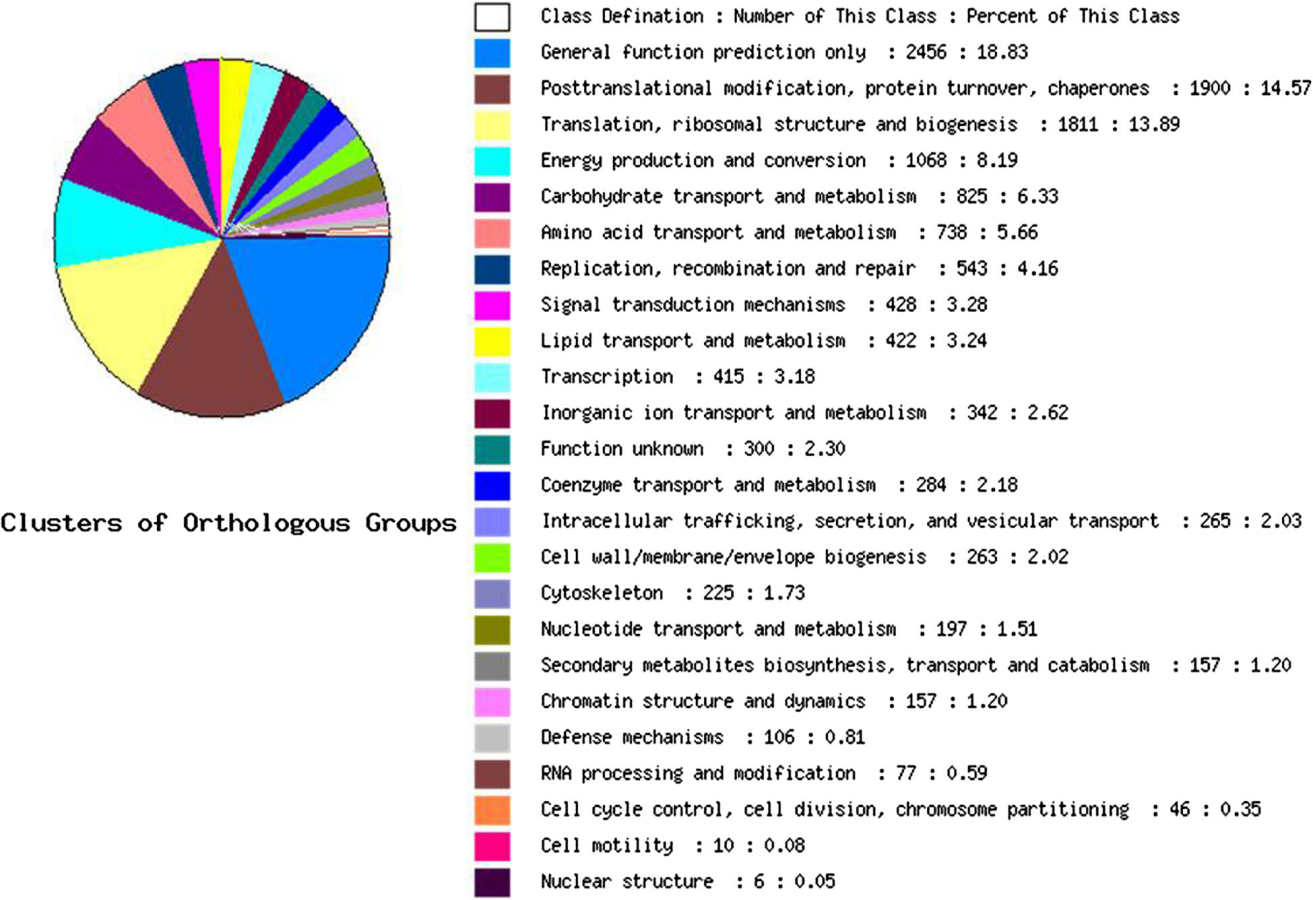
Clusters of orthologous groups (COG) classification.

The protein database Clusters of Orthologous Group (COG) has been designed to classify proteins from completely sequenced genomes based on orthology [27]. We searched annotated sequences for genes involved in COG classification to predict and classify the possible functions of all oriental persimmon unigenes. Of the 54,719 sequences that returned a hit with the Nr database, 14,954 could be assigned to 24 COG categories (Figure 4). Among these categories, the cluster for ‘general function prediction only’ was the largest group (2456, 18.83%), followed by ‘post-translational modification, protein turnover, chaperones’ (1900, 14.57%) and ‘translation, ribosomal structure and biogenesis’ (1811, 13.89%). The clusters for ‘nuclear structure’ (6, 0.05%), ‘cell motility’ (10, 0.08%), and ‘cell cycle control, cell division, chromosome partitioning’ (46, 0.35%) were the smallest groups.

**Figure 5:**
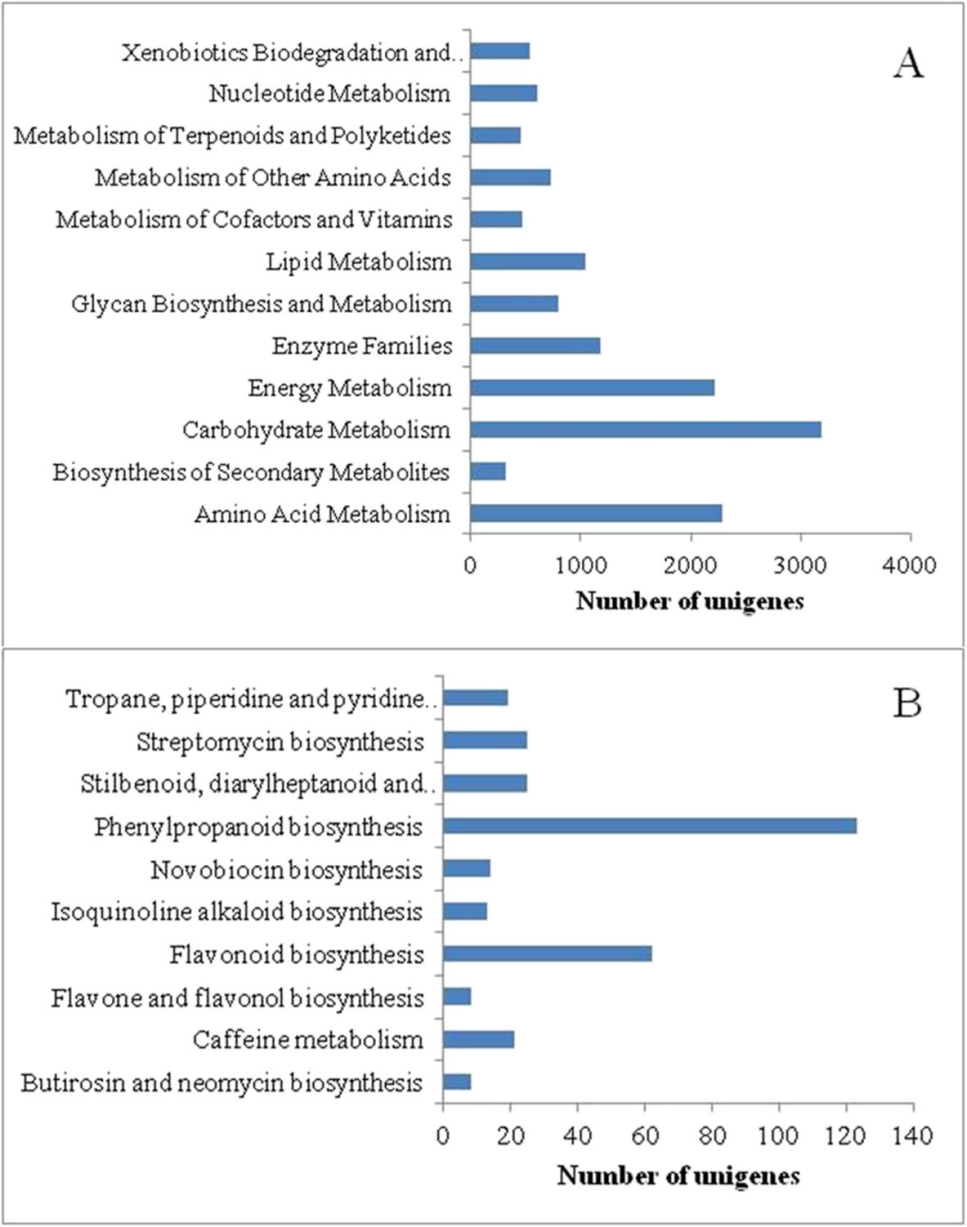
**Pathway assignment based on KEGG. (A)** Classification based on metabolism categories; **(B)** Classification based on secondary metabolism categories.

We mapped the annotated sequences to the reference canonical pathways contained in the KEGG database to identify the biological pathways in persimmons. This approach is an alternative way of categorising gene functions by emphasising biochemical pathways [28]. According to the KEGG results, 31,211 unigenes were mapped to 200 predicted metabolic pathways (Additional file 2). The transcripts identified as related to the following components or processes were the most abundant: translation (4638); folding, sorting, and degradation (4324); carbohydrate metabolism (3181); amino acid metabolism (2283); energy metabolism (2220); and transcription (1962). The largest category was metabolism (13,838), which included carbohydrate metabolism (3181), amino acid metabolism (2283), energy metabolism (2220), enzyme families (1179), lipid metabolism (1038), glycan biosynthesis and metabolism (804), metabolism of other amino acids (729), nucleotide metabolism (609), and other subcategories (Figure 5A). In the secondary metabolism category, 10 subcategories comprised 318 unigenes, the most represented of which were phenylpropanoid biosynthesis (123); flavonoid biosynthesis (62); stilbenoid, diarylheptanoid, and gingerol biosyntheses (25); streptomycin biosynthesis (25); caffeine metabolism (21); and tropane, piperidine, and pyridine alkaloid biosyntheses (19) (Figure 5B). In addition to the metabolic pathways, the genetic information processing pathways (12,838) and cellular processes pathways (2606) were also highly represented categories.

### Detection of differentially expressed unigenes in the Tr and Co libraries

A previous study proposed that comparing the number of reads for a gene between different libraries or different genes in the same library could be a reliable indicator of relative gene expression [29]. Thus, IDEG6 was used to identify unigenes that show a statistically significant difference in terms of relative abundance (as reflected by the total count of individual sequence reads) between the two libraries. A total of 3639 unigenes that were differentially expressed in the Tr and Co libraries were identified. Of these 3639 unigenes, 1560 were upregulated and 2079 were downregulated in the treated fruit. More genes were expressed in the treated fruit (15,331) than the control fruit (12,502) (Table 3).

**Table 3:** Summary of differentially expressed unigenes

	**Number of unigenes**	**Percentage (%)**
Total unigene	83898	
Differentially expressed in two library	3639	4.34
Up (Tr vs Co)	1560	1.86
Down (Tr vs Co)	2079	2.48
Expressed both in Tr and Co	56065	66.83
Expressed only in Tr	15331	18.27
Expressed only in Co	12502	14.9

### Quantitative real-time PCR confirmation

**Figure 6:**
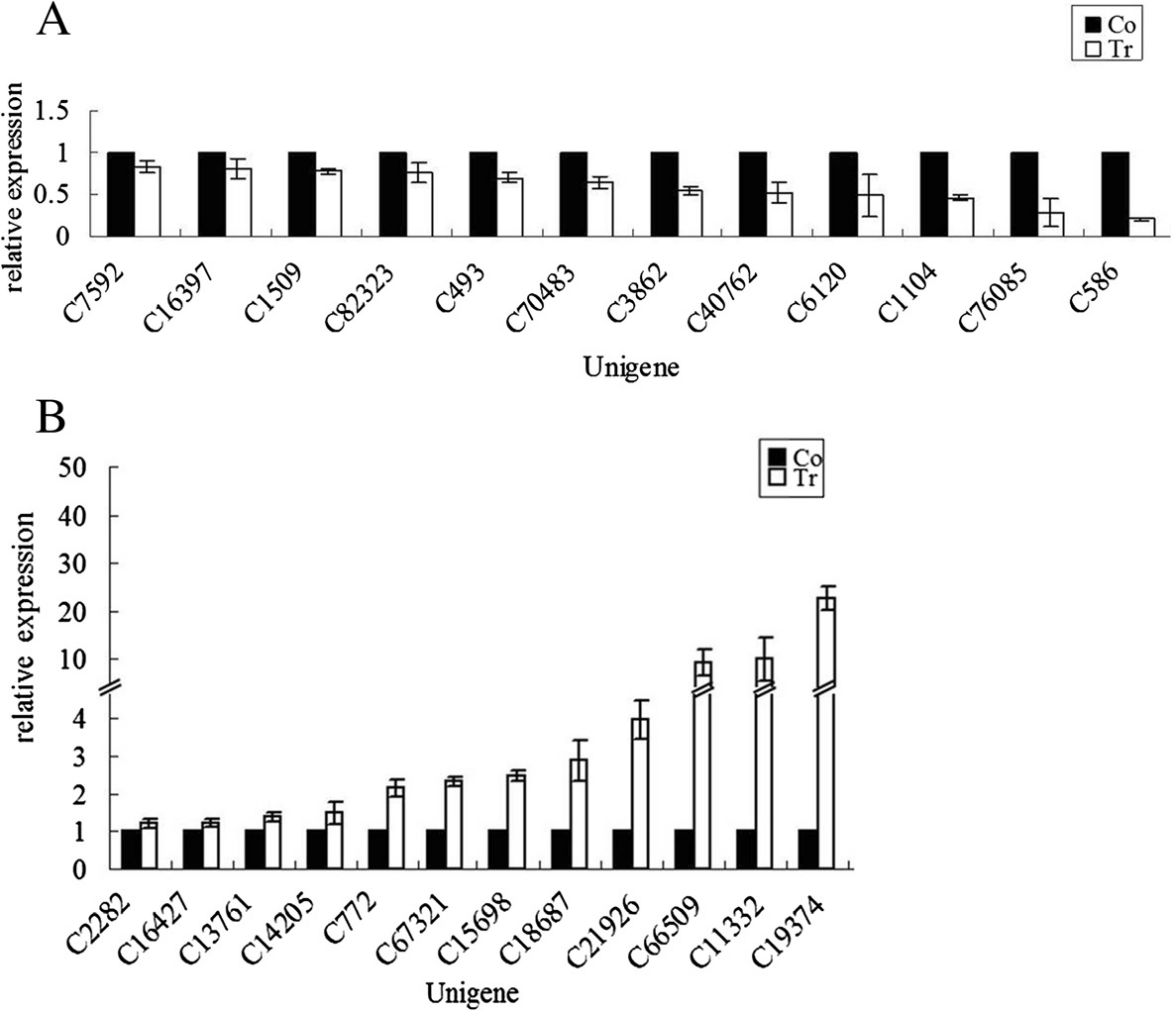
**Validation of part of differentially expressed unigenes by qRT-PCR. (A)** down-regulated unigenes in treated fruit. C7592: aldehyde dehydrogenase; C16397: aldehyde dehydrogenase family 2 member B7; C1509: NADH dehydrogenase; C82323: Transcription factor bHLH49; C493: 3-hydroxy-3-methylglutaryl-coenzyme A reductase 1; C70483: 3-dehydroquinate dehydratase/shikimate 5-dehydrogenase; C3862: *Diospyros kaki* anthocyanidin synthase; C40762: *D. kaki* flavonoid 3'5' hydroxylase; C6120: VTC2-like protein; C1104: *D. kaki* DkMyb2 mRNA for putative MYB transcription factor; C76085: *D. kaki* DKSCPL1 mRNA for serine carboxypeptitase-like protein 1; C586: DKSCPL1 mRNA for serine carboxypeptitase-like protein 1. **(B)** up-regulated unigenes in treated fruit. C2282: *D. kaki* MADS-box protein (MADS1) mRNA; C16427: dehydroascorbate reductase 1; C13761: aldehyde oxidase; C14205: *D. kaki* DkSK mRNA for putative shikimate kinase; C772: WD-repeat protein; C67321: 4-hydroxyphenylpyruvate dioxygenase; C15698: glutathione peroxidase; C18687: alcohol dehydrogenase (Adh3) mRNA; C21926: *D. kaki* beta-carotene hydroxylase mRNA; C66509: tetrahydroxychalcone 2'-glucosyltransferase; C11332: tonoplast intrinsic protein; C19374: ferulate 5-hydroxylase (F5H) mRNA.

Fifty differentially expressed unigenes were selected for qRT-PCR analysis (primers are shown in Additional file 3) to confirm the expression of the unigenes from the sequencing and computational analyses. cDNA fragments of the control and flesh treated for 3 d (15 July 2011; 73 d after bloom) were used as templates. The results showed that the qRT-PCR assessments (relative expressed level) of 34 unigenes (68%) were consistent with those of the 454 sequencing analysis (Figure 6). These results suggest that our transcriptome data have a high coverage.

## Discussion

Currently, the most common application of NGS in nonmodel species is transcriptome characterisation [30-34]. Among the currently available NGS technologies, 454 pyrosequencing produces the longest reads; thus, it has emerged as a powerful tool for transcriptome sequencing. In addition, many studies have used *de novo* assembly of such data to produce genome-level resources for non-model organisms [35-38].

Genetic studies of the persimmon are difficult to implement because of the hexaploid nature of the species and lack of linkage maps and whole genome sequences [i.e. only 14,189 EST sequences deposited in GenBank (accessed on 10/11/2013)]. Previous genetic studies have focused on the diversity and phylogeny of cultivated persimmons and related wild species. The transcriptome characterisation described in the present study will provide the initial information needed for the functional study of persimmons. A total of 624,690 and 626,203 reads were generated from the Tr and Co libraries, with average sequence lengths of 319 and 309 bp, respectively. We obtained 83,898 unigenes from these raw reads (Table 1). The average unigene length for persimmons in this study was comparable to that observed in other species such as *Oncidium* (493 bp) [39], *Pinus contorta* (500 bp) [40], *Fraxinus* (649 bp) [41], and *Vicia faba* (615 bp) [42]. Further, the unigene was longer than that observed in *Ziziphus celata* (408 bp) [43] and *Olea europaea* (355 bp) [44] but shorter than that in *Castanea mollissima* (731 bp) [31] and *Pyrus pyrifolia* (853 bp) [45]. The length of unigenes may be related to the sequencing technique and assembly tools used. However, the unigenes assembled in the present study could provide resources for future persimmon genetic and genomic research.

BLAST searches against public databases, such as NR, SwissProt, GO, and KEGG, provided annotation data for the persimmon, with 54,719 (65.2%) unique hits (Table 2). The annotation of persimmon gained more descriptive information than that observed in other species such as *Conyza canadensis* (51.3%) [46], *Bupleurum chinense* (52.6%) [47], *Dendrocalamus latiflorus* (54.9%) [48], *Lens culinaris* (55.6%) [49], and *Panax quinquefolius* (63.6%) [50]. However, it was lower than that observed in *Fagopyrum* (66.7%) [51], *Taxus cuspidata* (68.6%) [52], *Capsicum annuum* (72.04%) [53], *Olea europaea* (73%) [44], *Pyrus pyrifolia* (74.1%) [45], *Dendrocalamus latiflorus* (78.9%) [54], *Eichhornia paniculata* (87.0%) [55], and *Fraxinus* (99%) [41]. However, comparing this information across species studies is difficult because of the different sequencing depths or BLAST parameters utilized in each report [55].

A total of 31,211 persimmon unigenes were mapped into 200 KEGG pathways (Additional file 2). The genetic information processing pathway (12,838) and cellular processes pathway (2606) were highly represented categories in the metabolic pathways. Most persimmon fruits accumulate PAs in their flesh during development; PAs cause astringency due to coagulation of oral proteins and are synthesised from metabolites *via* the shikimate and flavonoid pathways [10-12]. Therefore, we focused on the pathways pertaining to phenylpropanoid biosynthesis (ko00940, 123 unigenes) and flavonoid biosynthesis (ko00941, 62 unigenes). Most of the genes related to PA biosynthesis in these two pathways were found in our transcriptome sequencing data. PAs cause astringency that is often undesirable for human consumption; thus, the removal of astringency is an important practice in the persimmon food industry. Soluble PAs can be converted to insoluble PAs by enclosing the fruit in a polyethylene bag containing diluted ethanol [56]. Acetaldehyde formed *in situ* from ethanol is involved in the direct insolubilisation of soluble PAs, causing a loss of astringency [57]. Pyruvate decarboxylase (PDC) and alcohol dehydrogenase (ADH) are two important enzymes in this process, which is involved in the glycolysis/gluconeogenesis pathway (ko00010, 496 unigenes). Moreover, six and 31 unigenes were classified into the PDC and ADH families, respectively.

The expression levels of the unigenes were reflected by the number of reads used to characterise the differences in gene expression between the Tr and Co transcriptome libraries. A total of 3639 unigenes were found to be differently expressed (Table 3). The expression levels of the unigenes involved in PA biosynthesis and tannin coagulation are shown in Table 4. The expression levels of *ADH1*, *4CL*, *ANS*, and *F3′5′H* were significantly downregulated, whereas those of *ADH3*, *PDC*, *CHS*, *F3H*, and *LAR* were significantly upregulated after the removal of astringency. These results suggest that the expression of genes involved in PA biosynthesis might be affected by ethanol treatment, which is consistent with the findings of Ikegami et al. [14]. Acetaldehyde produced from ethanol is involved in the direct insolubilisation of soluble PAs [57]. The synthesis of acetaldehyde is generally catalysed by PDC, which converts pyruvate to acetaldehyde; meanwhile, ADH is involved in the potentially reversible interconversion of ethanol and acetaldehyde [58]. Furthermore, *ADH1* and *PDC* are suggested as the key genes involved in persimmon astringency removal [59]. In the present study, the *PDC* gene was upregulated, which resulted in the production of more acetaldehyde. However, the *ADH1* gene was downregulated, which may result in the reduction of the conversion of acetaldehyde into ethanol. This result suggests that acetaldehyde accumulated in the ethanol-treated fruit, which resulted in the loss of astringency. However, this result was not consistent with that reported in Min’s study [59], where *ADH1* was upregulated by ethylene. This inconsistency in the results can be attributed to the different cultivars used (i.e. the non-astringent type ‘Luotian-tianshi’ was used in our study, while the astringent type ‘Mopanshi’ was used in Min’s research). The use of different materials might cause different gene expression patterns; however, this hypothesis needs to be validated by further experimentation.

**Table 4:** Summary of genes involved in PA biosynthesis and tannin coagulation

**Gene name**	**Number of unigenes**	**Number of reads/Co**	**Number of reads/Tr**
*ADH 1*	27	217	163
*ADH 2*	2	8	8
*ADH 3*	2	3	21
*PDC*	6	9	24
*ALDH2*	11	1,409	545
*PAL*	12	48	40
*4CL*	15	249	152
*CHS*	2	1	5
*CHI*	4	14	26
*F3H*	2	5	11
*F3’H*	2	5	8
*F3’5’H*	4	34	26
*DFR*	4	6	8
*LAR*	5	5	52
*ANS*	11	140	109
*ANR*	2	2	2
*MYB*	12	104	91
*bHLH*	3	86	90
*WD40*	2	14	32

We found that the gene aldehyde dehydrogenase family 2 (*ALDH2*) was highly expressed (1409 reads) in the Co library and downregulated in the Tr library (545 reads), with a total of 11 unigenes in both libraries (Table 4). ALDH2 has a broad expression pattern and is most notably involved in the second step of ethanol metabolism, (i.e. acetaldehyde oxidation). The decrease in *ALDH2* in the Tr library might have inhibited the conversion of acetaldehyde to acetic acid, which, consequently, led to acetaldehyde accumulation. Large amounts of acetaldehyde triggered the coagulation of tannins (insolubilisation of soluble PAs) causing the loss of astringency in the treated persimmon fruits. This result suggests that the *ALDH2* gene, together with the *ADH* and *PDC* genes, might have important functions in tannin coagulation.

The present study on persimmon transcriptome has several biological implications. First, the plant material persimmon, which accumulates PAs (condensed tannins) in its flesh during development, can be considered a model plant for tannin research. Second, the loss of astringency in CPCNA fruits treated with ethanol is an imitation of the natural loss of astringency, especially for tannin coagulation. This imitation helped us to understand mechanism of astringency loss in CPCNA. Third, the current study, based on the present transcriptome data (even in the absence of complete genome sequences for persimmons), will facilitate the advancement future genetic studies.

## Conclusions

This work presents the first *de novo* transcriptome sequencing analysis of the CPCNA persimmon fruit using the 454 GS FLX Titanium platform. A total of 374.6 Mb of data were generated and assembled into 83,898 unigenes. Persimmon unigenes related to PA biosynthesis were characterised, and differentially expressed unigenes in the two libraries were verified using qRT-PCR. *ADH*, *PDC*, and the newly discovered persimmon gene *ALDH2* were found to have important functions in tannin coagulation. To the best of our knowledge, this study is the first to employ the 454 sequencing technology to investigate the whole transcriptome of the persimmon fruit. The assembly of the reads was also conducted without a reference genome. The transcriptome characterisation described in the present study will provide the initial information needed for the functional study of persimmons to elucidate the molecular mechanisms of tannin coagulation and other biochemical processes in this fruit tree.

## Methods

### Sample preparation

In previous analysis of tannin concentration per fruit, JPCNA and CPCNA varied considerably. Both types accumulate PA in their fruits at an early stage. PA accumulation is halted in JPCNA at 7–9 weeks after bloom (WAB), and a low concentration of PA is observed at 10 WAB. On the other hand, CPCNA continuously accumulates PA until the late stages and maintains a very high PA concentration [22,23]. Thus, it appears that at 9–10 WAB, JPCNA and CPCNA exhibit different PA accumulation patterns. In the present study, 30 young fruits on a CPCNA-type persimmon tree (*D. kaki* ‘Luotian-tianshi’, 2n = 6X = 90) grown in the Persimmon Repository of Huazhong Agricultural University, China, were enclosed with polyethylene bags containing 10 mL of 5% ethanol on 12 July 2011 (10 WAB). Control (untreated) fruits were enclosed with polyethylene bags containing 10 mL of water. Three days later, all treated and control fruits were sampled and peeled. The flesh of the fruits was diced into small pieces, frozen in liquid nitrogen, and stored at −80°C until use for RNA isolation.

The concentrations of soluble and insoluble tannins in the control and treated fruit flesh were measured by the Folin–Ciocalteu method after 3 d of treatment [60]. Soluble tannins of the fruit flesh were also examined after 3 d of treatment by the printing method [61], which is a convenient way of identifying persimmon astringency loss. FeCl_2_ reacts with the soluble PAs; thus, the darker the resulting product, the more astringent the fruits.

### RNA extraction, cDNA library construction, and 454 sequencing

For each sample (5% ethanol treated and control), approximately 10 g of mixed flesh (10 individuals) was used for RNA preparation and tannin concentration measurements. Total RNA was extracted using TRIzol Reagent (Invitrogen, USA) following the manufacturer’s protocol. The quality and quantity of the total RNA was analysed using the NanoDrop 2000 spectrophotometer (Thermo Scientific, USA) and gel electrophoresis.

Approximately 1 μg of RNA was used to generate double-stranded cDNA using the SMART^TM^ cDNA Library Construction Kit (Clontech, USA). Finally, ~5 mg of cDNA was used to construct a 454 library. Roche GS-FLX 454 pyrosequencing was conducted by the Oebiotech Company in Shanghai, China.

### 454 *de novo* transcriptome assembly and analysis

A Perl program was written to remove vector sequences and the PolyA (T) tail from sequences; reads with lengths <100 bp were removed before assembly. Then, high-quality reads were assembled with MIRA [62] to construct unique consensus sequences. The 454 setting parameters were used by MIRA (−−job = denovo,est,normal, 454; -SK:mnr = yes; -SK:rt = 2; 454_SETTINGS -LR:mxti = no; -CL:qc = no).

The functions of the unigenes were annotated by BLASTX with an E-value threshold of 10^−5^ to the protein databases, including NCBI-NR, Swiss-Prot, KEGG [28], and COG [63]. InterPro domains [64] were annotated by InterProScan [65] Release 16.0, and functional assignments were mapped onto GO [66]. WEGO [67] was used for GO classification and GO tree construction.

### Differentially expressed unigene detection

A freely available web tool IDEG6 [68] was used to identify unigenes showing statistically significant differences in relative abundance (as reflected by the total count of individual sequence reads) between the Tr and Co libraries. The general Chi-squared method was used because it was the most efficient analytical method [68]. Finally, unigenes with P ≤ 0.01 were deemed significantly different between the two libraries.

### RNA extraction, first-strand cDNA synthesis, and qRT-PCR analysis

A total of 50 unigenes generated by 454 sequencing were selected for experimental validation. The total RNA used for qRT-PCR analysis was extracted from the flesh of the Tr and Co fruits. After RNA extraction, first-strand cDNA was synthesised from 1.0 μg of RNA using the PrimeScript® RT Reagent Kit with gDNA Eraser (TaKaRa, Dalian, China) according to the manufacturer’s protocol. The cDNA was diluted threefold and used as the template for qRT-PCR. qRT-PCR was performed on a LightCycler® 480 II System (Roche Diagnostics) using SYBR® *Premix Ex Taq*^TM^ II (TaKaRa). The reaction was composed as described in the manual and was performed in quadruplicate. A negative control (no template) was included in each run. The standard amplification protocol consisted of an initial denaturing step of 95°C for 30 s, followed by 45 cycles of 95°C for 5 s, 60°C for 10 s, 72°C for 15 s, and a melting temperature cycle with constant fluorescence data acquisition from 65°C to 95°C. The gene quantification method was based on the relative expression of the target gene versus the reference gene (*DkActin*), and the ratio was calculated with the LightCycler® 480 software. All primers are listed in Additional file 3.

## Competing interests

The authors declare that they have no competing interests.

## Authors’ contributions

Conceived and designed the experiments: L-ZR, LC and Z-QL. Performed the experiments and analyzed the data: LC. Wrote the manuscript: LC. Read and approved the final manuscript: LC, Z-QL and L-ZR.

## Supplementary Material

Additional file 1 GO classifications of CPCNA persimmon unigenes. Click here for file

Additional file 2 KEGG pathway annotation of CPCNA persimmon unigenes. Click here for file

Additional file 3 **Information for primers used in qRT-PCR analysis.** The primer sequences of 34 unigenes verified successfully by qRT-PCR were listed in this table. Click here for file
