# Supplementary material for: Genome-wide transcriptome analysis of Chinese pollination-constant nonastringent persimmon fruit treated with ethanol
Source: BMC Genomics. 2014 Feb 8;15:112. doi: 10.1186/1471-2164-15-112 (PMC3922992; doi:10.1186/1471-2164-15-112)
Supplement: Additional file 3 — Information for primers used in qRT-PCR analysis. The primer sequences of 34 unigenes verified successfully by qRT-PCR were listed in this table. [file 1471-2164-15-112-S3.doc]

Additional file 3 Information for primers used in qRT-PCR analysis.

| Unigene | Sequence (5’-3’) | Tm (°C) |
| --- | --- | --- |
| C7592 | F: CGCCTTACGAGCAGCAGA  R: GGAACCGATTGTCCCACC | 53.6 |
| C16397 | F: AGGAACCGATTGTCCCACC  R: CACCCTCAGCAACATTAGCATA | 52.7 |
| C1509 | F: GTTCTCGCTCAGTGTTTGC  R: GGTCGTGCGTGGTATGTT | 52.8 |
| C82323 | F: TTCAGCCACATTCACCTC  R: GTTTATCTCAGTTTCCAGCA | 50.0 |
| C493 | F: CCTGGGTGACATCTTTGG  R: TGAACCTGCTGGGAGTGA | 53.3 |
| C70483 | F: CCAGCACCAATGACAACA  R: GCACTGCGAGGACTAAAT | 51.0 |
| C3862 | F: GCTTGTTGCTGGGATGTAA  R: GCGTGAATGGGAGGACTA | 50.8 |
| C40762 | F: AATGCCCATCCTAACTCC  R: GCTACTACATACCCAAGAACAC | 51.2 |
| C6120 | F: GAACCCATACTTCCGACTG  R: TAAGCACATTGTAAGGCAC | 50.4 |
| C1104 | F: TCCTGCGGGATGACTACTTT  R: GGGCGAACAGACAATGAGAT | 52.5 |
| C76085 | F: TACTGTTCATAGTGCCCTCTT  R: TTCACGGACTCCCTCATT | 50.0 |
| C586 | F: CACTGTTCCTCTGCTCGTT  R: ACTCACTTCCGCATCCTC | 50.0 |
| C2282 | F: CTTTGGCTGATCGGTTAT  R: AAGAGGAGGAATGGGTTG | 50.0 |
| C16427 | F: CATTGACCTTTGGCTCCC  R: TTTCTGCGGTTGATTTGA | 50.9 |
| C13761 | F: GAAATGAAAGGAGGGACA  R: GCAGATATGGCAGCAGAG | 50.0 |
| C14205 | F: GTATGACTGAGTAAAGGAGCAC  R: ACGGGAACAGAAGTAGCG | 51.1 |
| C772 | F: ACCCAAGGTGACTATGAC  R: CACCACCCAGTAAACTCC | 51.0 |
| C67321 | F: CCGCCTTTCTGATATTCT  R: TTTGGTGGACAGGGATGA | 50.5 |
| C15698 | F: GCGGAGCAGTAGTAGGAG  R: AATGTGAACGGTGATGGT | 50.0 |
| C18687 | F: GGGGTTCCCATTTATTGA  R: AGACGCCATTGTTTCCTC | 52.2 |
| C21926 | F: CCCGCTCCTTCATCCATA  R: TTCCTTCACCACGAAACAGA | 55.1 |
| C66509 | F: ACACGGAGGTAGTGAAGG  R: TATCTATGGGTGGACAAG | 51.2 |
| C11332 | F: TGTTGGGTTGTATGATTCGC  R: CCCGTCTATGGCTTTGATTT | 50.2 |
| C19374 | F: AACCCAAATGTGACACGA  R: GCAAAGAGCTGGAAAGATAA | 50.0 |
| C65499 | F: AGTCGTTGCTGTTAGGGTT  R: GGTCACGCCATTTTCTTAT | 50.5 |
| C12835 | F: TGACAAACCAATACAGCAGG  R: TTGACCAGAAGCAGCGAC | 50.8 |
| C24350 | F: GGAGAAACGACTCTTGCG  R: CTGAGGTTCATCCTGGTG | 50.0 |
| C66622 | F: AGACCATCTGGGTAACAACT  R: CTTCTTCGGCAACTTCAA | 50.8 |
| C1606 | F: TCTTGTAGTGCTCCTTTGTG  R: GGCTTCTTTGAGGTGGTG | 50.0 |
| C1743 | F: GCCCTCACTTTCAATCCA  R: TAGCCCACAAGACATCCC | 50.2 |
| C19828 | F: TCACGAGCCTTTGGATAA  R: CATAACGGTGTTTGGGAT | 52.8 |
| C203 | F: GGTTCCATCCACGGGCTCA  R: TTGGTCGATTGTCGTTCTCCTT | 51.9 |
| C641 | F: AGGCACAGTTACAGTCAGC  R: AGGAGCAAGGAGCAGGAG | 52.6 |
| C673 | F: TCAGGTTCCGCAGCAGCAT  R: GGACCCTACCAGTGATCCAA | 56 |
